# Shifts from cooperative to individual-based predation defense determine microbial predator-prey dynamics

**DOI:** 10.1038/s41396-023-01381-5

**Published:** 2023-02-28

**Authors:** Magali de la Cruz Barron, Ellen van Velzen, Uli Klümper, Markus Weitere, Thomas U. Berendonk, David Kneis

**Affiliations:** 1 Helmholtz Centre for Environmental Research—UFZ, Department of River Ecology, 39114 Magdeburg, Germany; 2 TU Dresden, Institute of Hydrobiology, 01062 Dresden, Germany; 3 University of Potsdam, Institute of Biology and Biochemistry, 14469 Potsdam, Germany

**Keywords:** Bacterial evolution, Population dynamics, Theoretical ecology

## Abstract

Predation defense is an important feature of predator-prey interactions adding complexity to ecosystem dynamics. Prey organisms have developed various strategies to escape predation which differ in mode (elude vs. attack), reversibility (inducible vs. permanent), and scope (individual vs. cooperative defenses). While the mechanisms and controls of many singular defenses are well understood, important ecological and evolutionary facets impacting long-term predator-prey dynamics remain underexplored. This pertains especially to trade-offs and interactions between alternative defenses occurring in prey populations evolving under predation pressure. Here, we explored the dynamics of a microbial predator-prey system consisting of bacterivorous flagellates (*Poteriospumella lacustris*) feeding on *Pseudomonas putida*. Within five weeks of co-cultivation corresponding to about 35 predator generations, we observed a consistent succession of bacterial defenses in all replicates (*n* = 16). Initially, bacteria expressed a highly effective cooperative defense based on toxic metabolites, which brought predators close to extinction. This initial strategy, however, was consistently superseded by a second mechanism of predation defense emerging via de novo mutations. Combining experiments with mathematical modeling, we demonstrate how this succession of defenses is driven by the maximization of individual rather than population benefits, highlighting the role of rapid evolution in the breakdown of social cooperation.

## Introduction

Classical theory predicts predator-prey systems to develop toward an equilibrium where species abundances undergo regular oscillations [1, 2] or coexist in a steady-state [3]. While this notion has been confirmed experimentally [4], the dynamics of many real-world systems hardly conform to these predictions even when external perturbations are absent. A considerable amount of complexity is added to predator-prey dynamics through the appearance of grazing defense strategies [5, 6]. Especially for bacteria exposed to protozoan grazing, a variety of alternative defenses which can interrupt oscillations and entail shifts in predator-prey ratios have been described [7]. Known bacterial defenses can be classified along multiple criteria. Considering the mode of action, certain active defenses represent a form of attack, e.g. predator poisoning [8–10], while passive strategies rather reduce the risk of capture or ingestion through morphological alteration [11, 12] or aggregation [13, 14]. Taking the scope of protection as a criterion, strategies conferring grazing resistance of prey individuals are in contrast to community defenses with the latter relying on social cooperation [15]. Although community defenses like, e.g., the production of extracellular toxic compounds, are very efficient at high bacterial densities [8, 16], they remain vulnerable to “cheating” [17]. Reversibility of a defense is yet another criterion for classification: Inducible phenotypic adaptations [11, 14] can be distinguished from permanently grazing protected genotypes emerging from de novo mutations [18–20] or selection on standing genetic variation [21–24].

Most previous experiments focused on singular mechanisms of prey defense typically observable within hours to a couple of days [8, 9, 13, 14, 25, 26]. While these studies improved our mechanistic understanding of particular defenses substantially, important ecological and evolutionary facets impacting long-term predator-prey dynamics remained largely unexplored. This pertains especially to the recognition of multiple defenses in one and the same prey strain, the possible interaction between alternative defenses, and evolutionary optimization of strategies toward improved protection or cost amelioration. The role of evolution in particular remains poorly understood since only few studies managed to trace down grazing resistance to spontaneously occurring beneficial mutations in replicate experiments [27, 28].

In this study, we followed the dynamics and traits of prey and predators in semi-continuous, planktonic cultures over periods of five weeks. Specifically, *Pseudomonas putida* was co-cultured with the bacterivorous nanoflagellate *Poteriospumella lacustris* under conditions of daily dilution and resource replenishment to study prey defenses over many generations.

Within the period of five weeks, we observed a unique but consistent succession of bacterial defense strategies, including the evolution of new phenotypes, across all of the 16 replicate co-cultures. In all replicates, a successful initial defense that brought predators close to extinction was consistently superseded by a second defense, with the latter being inferior in terms of reducing predator abundance. This succession of defenses was *not* driven by an invalidation of the original defense due to predator adaptation.

Here, we demonstrate the mechanisms behind this counter-intuitive succession of bacterial defenses by combining dedicated experiments with dynamic ecological modeling. We elucidate triggers, costs, and benefits of the two defenses to disclose the (eco)-logic underlying their succession. We also demonstrate that successful cooperative defenses are not only vulnerable to social cheating but are even more susceptible to the emergence of individual-based protection conferred by rare favorable mutations that evolve rapidly.

## Materials and methods

### Organisms and culture medium

The examined predator-prey system consisted of *Pseudomonas putida* strain KT2440 and the bacterivorous spumella-like flagellate *Poteriospumella lacustris* strain JBM 10. During experiments, the two organisms were cultivated in wheat grass medium [29]. Briefly, a concentrated infusion was prepared from wheat grass (GSE Vertrieb GmbH, Germany) [30] at a concentration of 25 g L^−1^ which was subsequently cleared from larger particles through filtration (0.2 µm pore size). 10 mL L^−1^ of the concentrated infusion were supplemented with a balanced salt solution (1.78 mM NaCl, 0.42 mM MgCl_2_, 0.16 mM MgSO_4_, 0.31 mM KCl and 0.09 mM CaCl_2_, as final concentrations), NH_4_Cl (100 mg L^−1^) and phosphate buffer (pH 7, Na_2_HPO_4_ x 2 H_2_O at 500 mg L^−1^ and NaH_2_PO_4_ x H_2_O at 120 mg L^−1^). Contrary to the original recipe [29], no stigmasterol was added. The medium was autoclaved for 20 min at 121 °C.

### General experimental setup

Bacterial defenses to flagellate predation were explored in semi-continuous cultures experiencing a dilution rate of 0.5 day^−1^. That is, every day, 50% of the culture volume was transferred to a new sterile vial replenished with an identical volume of fresh wheat grass medium. In that way, the formation of mature bacterial biofilm, which is one of the most efficient means to acquire protection from grazing by planktonic nanoflagellates [14, 25], was suppressed entirely.

All experiments were performed in 100 mL glass vials filled with only 10 mL of cell culture to prevent notable oxygen depletion. Vials were incubated at 19 °C on a rotary shaker (120 rpm). Samples were taken every 24 h to quantify cell densities by microscopy.

### Initial conditions and replication strategy

Experiments were started with different initial conditions regarding prey and predator densities. We expected that varying predation pressure levels might lead to different defense dynamics, because it is known that the expression of prey defenses can be predator density-dependent [31]. Besides two experimental lines with low and high initial flagellate densities, experiments without any predators were performed for control purposes (Table 1).

**Table 1:** Initial predator and prey densities used in different experimental lines.

Predation pressure	Flagellates mL^−1^	Bacteria mL^−1^
Initially high	1 × 10^5^	1 × 10^5^
Initially low	1 × 10^3^	1 × 10^4^
Absent throughout (control)	0	1 × 10^4^

The bacteria used for inoculation were grown overnight in wheat grass medium (19 °C, shaken at 120 rpm) to resemble the conditions in the actual experiments. All bacterial cultures were started from a single colony of *P. putida* (plated on LB) to guard against initial genotypic heterogeneity. The flagellate inoculum was obtained from axenic stock cultures held in NSY medium (3 g L^−1^) at 16 °C. To avoid significant carryover of nutrients from stock cultures, flagellates were washed twice in wheat grass medium (10 min centrifugation at 3000 rpm). Both bacteria and flagellate pre-cultures were diluted as necessary to obtain the initial densities listed in Table 1 upon mixing with 9.5 mL of wheat grass infusion.

Temporally independent experiments considering all initial conditions listed in Table 1 were started at three points in time (September and October 2020; April 2021). Additional experiments with an inoculum of 1 × 10^5^ flagellates mL^−1^ were run in April and June 2021. Independently of the initial conditions and starting date, all experiments were run in duplicates.

### Quantification of cell abundances

**Fig. 1:**
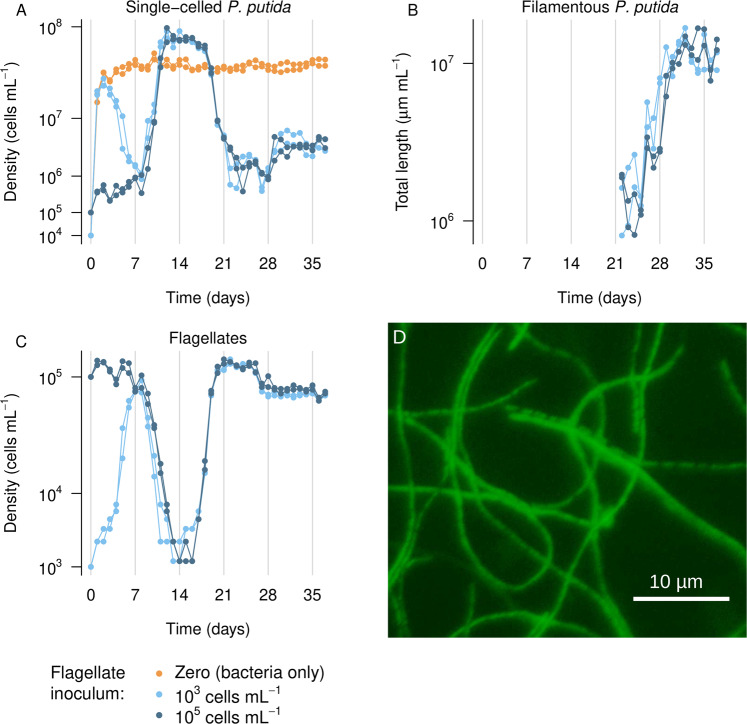
Observed predator-prey dynamics over 5 weeks under different initial conditions. **A**–**C** Abundance of single-celled and filamentous *Pseudomonas putida* and flagellates (*Poteriospumella lacustris*). Dots represent individual observations; lines highlight their association with a particular replicate. Scenarios with different initial conditions are distinguished by light and dark blue color. The coefficient of variation for replicate observations never exceeded a value of 0.5 for any time point after day six. **D** Microscope image of bacterial filaments stained with nucleic acid-specific dye SYBR Green I.

Predator and prey abundances were quantified daily in all replicates. Prey numbers were determined after bacterial staining with SYBR Green I Nucleic Acid Gel Stain (S7563, Invitrogen) at a final concentration of 1X for 15 min in the dark. Samples were filtered onto 0.2 µm pore size black polycarbonate filters (Whatman 7063-2502) and counted by epifluorescence microscopy. At least 250 individuals at randomly selected spots were counted per sample. Filament length and size distribution were recorded in the four replicates from October 2020 (Fig. 1A–C). The length of 100 randomly selected individuals was measured per sample and day. All cell measurements and bacterial counts were performed using the NSI-elements AR 5.11.01 software. Predator density was estimated, in technical duplicates, by means of Neubauer improved counting chambers (C-chip Neubauer Improved DHC-N01 chambers, NanoEntek), following manufacturer instructions and using 10 µL as loading volume.

### Identification of mutations in filamentous bacteria

Nine filamentous isolates obtained from individual replicates and a non-filamentous control isolate were whole-genome sequenced at Eurofins Genomics (Ebersberg, Germany). Bacterial cells were obtained by centrifugation of 1 mL of an overnight culture of each isolate. Thereafter, DNA was extracted using the DNeasy Powersoil kit (Qiagen) according to the manufacturer’s instructions. Sequencing libraries were prepared using the Illumina standard genomic library protocol, and sequenced on a NovaSeq 6000 (Illumina) using the 2 × 150 bp paired-end read mode, resulting in approximately 5 million paired reads and a minimum coverage of 100-fold.

The obtained sequences were quality checked with windowed adaptive trimming in SICKLE [32]. High quality sequences were then mapped against the published KT2440 reference genome (https://www.ncbi.nlm.nih.gov/assembly/GCA_000007565.2) using BWA-MEM [33]. A consensus genome was then called from the output using Ococo [34]. The respective pipeline was implemented using the Galaxy platform [35]. Nucleotide mismatches between the genomes of filamentous isolates and a non-filamentous control were located and associated with coding sequences identified by Prokka [36]. Nucleotide sequences of interest were translated into peptide sequences with EMBOSS Transseq [37] to distinguish synonymous and non-synonymous base substitutions. Sequences have been uploaded to the NCBI SRA database (accession number: PRJNA830374).

### Verification of flagellate growth inhibition by bacterial metabolites

To verify the inhibition of predators by bacterial metabolites, we examined the growth rates of axenically grown flagellates when confronted with filtrates harvested from co-cultures (bacteria + flagellates) and control cultures (bacteria only). Likewise, flagellate populations which had previously undergone recovery from metabolite-induced growth inhibition were again exposed to toxic metabolites to test for possible adaptation effects.

The filtrates employed for the tests were obtained from co-cultures and predator-free control cultures by sterile filtration using syringe filters of 0.2 µm pore size. Filtrates were harvested at different experimental phases: after launch (day 2–3), during the collapse of the predator population when growth inhibition was most apparent (day 8–12), and from the steady state (day 27–29). All filtrates were stored at 4 °C until use.

Before the exposure tests, axenically grown flagellates were washed by centrifugation, suspended in sterile Volvic water and cultured with washed *P. putida* as a food source (2 × 10^7^ cells mL^−1^) for 24 h. Flagellate densities were quantified as previously described, and adjusted to the cell densities needed for exposure tests.

The flagellates present in co-cultures were obtained after their recovery at the onset of the filamentous phase (day 18–21). On the day of the exposure test, flagellates were harvested by centrifugation (10 min centrifugation at 3000 rpm), suspended in sterile Volvic water, quantified, and adjusted to the cell densities needed for exposure tests.

Exposure to filtrates from either co-cultures or control cultures was performed in triplicate batches of 3 mL volume each. Irrespective of the origin of filtrate and flagellates, all replicates containing 2.7 mL of filtrate were inoculated with 0.3 mL of flagellate suspension with minor variation in concentrations from day to day (11,250 to 52,500 individuals mL^−1^). Cultures were continuously shaken at 120 rpm at 19 °C. Flagellate densities were recorded after 24 h of exposure and growth rates were computed considering the respective initial values.

### Mathematical modeling

A model rooted in ordinary differential equations (ODE) was implemented in R/Fortran [38] to simulate the dynamics of predator and prey. Numerical integration of the simultaneous ODE was paused every 24 h and all state variables were updated to reflect the daily transfer of 50% of the culture volume to a new flask with fresh sterile medium. The semi-continuous model accounts for seven state variables, four of which represent *Pseudomonas putida* in distinct phenotypes or life stages, respectively (Table 2). Observation data exist for a subset of variables, namely flagellates (*F*), bacterial filaments (*Bff*), and the total abundance of single-celled bacteria (sum of *Bo*, *Bx*, and *Bfs*). Both data and model are accessible via the git repository https://github.com/dkneis/KT2440defense.

**Table 2:** State variables of the dynamic model.

Symbol	Description	Unit
*R*	Concentration of resources for bacterial growth.	µg mL^−1^
*Bo*	Abundance of undefended *P. putida* cells.	cells mL^−1^
*Bx*	Abundance of single-cell *P. putida* excreting secondary metabolites. The latter are represented by state variable *X*.	cells mL^−1^
*Bff*	Abundance of filamentous *P. putida*. A section of 1 µm length was considered a single-cell equivalent.	cell equivalents mL^−1^
*Bfs*	Abundance of single-celled *P. putida* belonging to the filamentous genotype. This type of cell results from asymmetric division of filaments (state variable *Bff*).	cells mL^−1^
*F*	Abundance of the flagellate *Poteriospumella lacustris* feeding on single-celled bacteria.	cells mL^−1^
*X*	Concentration of secondary metabolites excreted by the bacterial phenotype *Bx*. The metabolites enhance resource exploitation by bacteria and inhibit flagellate growth.	mass mL^−1^

See Table 1 for the respective initial values.

Following the philosophy of the rodeo [38] package, the ODE were compiled in the form of Eq. (1) to relate the derivatives of the *n* state variables (vector *y*; cf. Table 2) to the action of *k* distinct processes (transposed vector *r*) in a transparent and computationally efficient manner. The linkage between states and processes is accomplished through a *k* × *n* matrix of stoichiometric factors denoted *Q* taking into account constant parameters *u*.

1 $$\frac{d}{{dt}}y = r\left( {y,u} \right)^T \cdot \,Q\left( {y,u} \right)$$

**Fig. 2:**
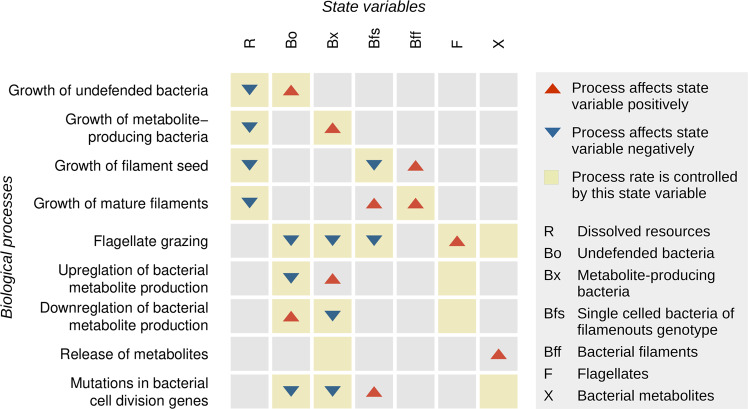
Qualitative stoichiometric matrix illustrating the impact of biological processes on the state variables of the ODE-based model. Note that the intermittent, purely physical process of resource replenishment associated with a dilution of all other constituents is not depicted. See Table 3 for the numerical representation of the matrix.

The entire set of simultaneous ODE expressed in terms of *r* and *Q* with *n* = 7, *k* = 9 is specified in Table 3. The full right hand side expression corresponding to the derivative of a particular state variable is obtained by multiplying the vector of process rate expressions (*r*, second column of Table 3) with the respective column of the stoichiometry matrix (shaded part of Table 3). A graphical outline of the model based on a qualitative representation of the stoichiometry matrix *Q* can be found in Fig. 2. The rationales for the particular formulations of process rates and stoichiometric factors presented in Table 3 are outlined below.

**Table 3:** Expressions of process rates and stoichiometric factors used to simulate the dynamics of all state variables from Table 2 between distinct transfer events.

		Stoichiometry matrix *Q (State variables as column headers)*						
Process	Process rate expressions (vector *r*)	*R*	*Bo*	*Bx*	*Bfs*	*Bff*	*F*	*X*
Bacterial growth (*Bo*)	*m · monod(R, hB, zB) · Bo*	*α*	*1*					
Bacterial growth (*Bx*)	*m · cBx · monod(R, hB, zB) · Bx*	*α*		*1*				
Bacterial growth (*Bfs*)	*m · cBf · monod(R, hB, zB) · Bfs*	*α*			*−1*	*1*		
Bacterial growth (*Bff*)	*m · cBf · monod(R, hB, zB) · Bff*	*α*			*p*	*1-p*		
Flagellate grazing	*g · monod(Bo* + *Bx* + *Bfs, hF, zF) · F · inh(X, micX)*		*-Bo/β*	*-Bx/β*	*-Bfs/β*		*yF*	
Upregulation of metabolite production	*ku · Bo · on(Bo, tuB) · on(F, tuF)*		*−1*	*1*				
Downregulation of metabolite production	*kd · Bx · max(off(Bo, tuB), off(F, tuF))*		*1*	*−1*				
Excretion of metabolites	*kx · Bx*							*1*
Filamentation	*kf · (Bx* + *Bo) · on(X, tf)*		*-Bo/γ*	*-Bx/γ*	*1*			

See Tables 4 and 5 for the declaration of additional symbols representing functions and parameters, respectively. Greek symbols encode auxiliary expressions as follows: *α* = *−1 / (yB · (1* + *iyB · on(X, ty)))*; *β* = *Bo* + *Bx* + *Bfs*; *γ* = *Bo* + *Bx*. See Fig. 4 for a graphical representation of the simultaneous ODE.

#### Growth of bacterial fraction *Bo*

Represents the growth of undefended single-cell bacteria. The expression is very common except for the stoichiometric factor *α*. The latter accounts for the fact that the yield (amount of bacterial cells produced from a certain amount of resource, symbol *yB*) increases when secondary metabolites (*X*) exceed a certain threshold (see function *on()*, Table 4).

**Table 4:** Definition of parameters appearing in process rate expressions and stoichiometric factors presented in Table 3.

Symbol	Value	Unit	Description
*m*	2	h^−1^	Max. growth rate constant of bacteria
*hB*	344	µg mL^−1^	Monod constant for bacterial growth on resource *R*
*zB*	2	µg mL^−1^	Minimum resource level for bacterial growth
*g*	20	cells h^−1^	Maximum number of single-celled bacteria ingested by flagellate individuals
*hF*	1100,000	cells mL^−1^	Monod constant for flagellate ingestion
*zF*	450,000	cells mL^−1^	Minimum prey density for flagellate ingestion
*yB*	430,000	cells µg^−1^	Yield of bacteria growing on resource *R* when ambient metabolite levels are low
*iyB*	1	–	Relative increase in bacterial yield at high ambient metabolite concentrations
*yF*	0.0028	cells cells^−1^	Flagellate cells produced per ingested bacterial cells
*tuF*	60,000	cells mL^−1^	Critical flagellate density to trigger upregulation of metabolite production
*tuB*	980,000	cells mL^−1^	Critical bacterial density to trigger upregulation of metabolite production
*tf*	8,000,000	mass mL^−1^	Critical metabolite concentration to trigger filamentation
*ty*	14,400,000	mass mL^−1^	Metabolite concentration above which bacterial yield is increased
*micX*	24,400,000	mass mL^−1^	Minimum metabolite concentration to suppress flagellate growth
*ku*	0.009	h^−1^	Controls the upregulation of gene(s) mediating metabolite production
*kd*	0.037	h^−1^	Controls the downregulation of gene(s) mediating metabolite production
*kf*	0.0005	h^−1^	Controls the rate of bacterial mutations resulting in filamentation
*p*	0.6	–	Probability of asymmetric division during filament growth
*kx*	1	mass cell^−1^ h^-1^	Controls the amount of metabolites excreted by bacteria
*cBx*	0.89	–	Fitness cost associated with metabolite production
*cBf*	0.95	–	Fitness cost associated with filamentation

#### Growth of bacterial fraction *Bx*

Represents the growth of metabolite producing single-cell bacteria. Very similar to the growth expression for *Bo*, but a factor (*cBx* < *1*) was added to account for a fitness disadvantage arising from metabolite production.

#### Growth of bacterial fraction *Bfs*

Represents the transition from single-celled mutants to actually filamentous individuals. The factor *cBf* accounts for fitness costs associated with the filamentous trait.

#### Growth of bacterial fraction *Bff*

Represents the growth of bacterial filaments. The factor *cBf* accounts for fitness costs associated with the filamentous trait. The stoichiometric factors *p* and *1-p* account for the fact that filaments elongate or divide asymmetrically with certain probabilities. Division adds to the pool of single-celled filament offspring (*Bfs*).

#### Flagellate grazing

Represents the elimination of single-cell bacteria by grazing. The actual elimination rate is obtained by correcting an intrinsic maximum ingestion rate of flagellates for the effects of prey density (Monod model) and the ambient concentration of bacterial secondary metabolites (inhibition model of MIC type). The stoichiometric factors involving *β* account for the proportional elimination of prey phenotypes.

#### Upregulation of metabolite production

Describes the upregulation of gene(s) involved in the production of secondary metabolites by single-celled bacteria. Upregulation is represented as a flux from state variable *Bo* to *Bx* which occurs when the abundance of bacteria and predators simultaneously exceed certain thresholds (predator sensing combined with quorum sensing).

#### Downregulation of metabolite production

Downregulation is modeled as the reverse of upregulation, i.e. as a flux from *Bx* to *Bo*. It occurs as soon as either of the two conditions for upregulation is no longer met.

#### Excretion of metabolites

Represents the excretion of secondary metabolites by cells with upregulated expression of the responsible gene(s).

#### Filamentation

Represents the switch from a single-celled to a filamentous bacterial genotype due to mutation. This is modeled as a first-order conversion process with a very small rate constant. It is assumed that mutations preferentially emerge at elevated metabolite concentrations indicating bacterial stress.

Because several of the processes are controlled by threshold values, the model exhibits a strongly non-linear behavior. Consequently, simulation results are exceptionally sensitive to changes in the values of some of the parameters (Table 5). The most sensitive ones include the minimum concentration of bacterial metabolites causing an inhibition of flagellate growth (*micX*), the threshold organism densities triggering metabolite production (*tuF, tuB*), but also the yield coefficient of flagellates (*yF*).

**Table 5:** Definition of functions appearing in process rate expressions and stoichiometric factors from Table 3.

Symbol	Description
*monod*	Monod model with the first argument (*x*) being a concentration or abundance and the second argument (*h*) being a half saturation constant. The non-standard third argument (*z*) is a horizontal shift parameter. The latter represents a minimum concentration / abundance below which the function returns zero. $$monod(x,h,z) = \left\{ {\begin{array}{*{20}{c}} 0 & {{{{{{{{\mathrm{for}}}}}}}}\,x - z \le 0} \\ {{\textstyle{{x \, - \, z} \over {x \, - \, z \, + \, h}}}} & {{{{{{{{\mathrm{else}}}}}}}}} \end{array}} \right.$$
*inh*	Standard linear inhibition model. The first argument is the concentration of the inhibitor (*x*), the second argument is the minimum inhibitory concentration (*mic*). $$inh(x,mic) = \left\{ {\begin{array}{*{20}{c}} 0 & {{{{{{{{\mathrm{for}}}}}}}}\,x \ge mic} \\ {1 - {\textstyle{x \over {mic}}}} & {{{{{{{{\mathrm{else}}}}}}}}} \end{array}} \right.$$
*on*	Steep sigmoidal function to represent an “upward” step. Returns zero if the first argument (*x*) falls below a threshold defined by the second argument (*t*), otherwise returns one. The coefficients *a* and *b* controlling the steepness were chosen to be *a* = *0.9 t* and *b* = *1.1 t*. $$on(x,t) = \left\{ {\begin{array}{*{20}{c}} 0 \\ {{\textstyle{1 \over 2}}} \\ 1 \\ 1 \end{array}\begin{array}{*{20}{c}} {} & {{{{{{{{\mathrm{for}}}}}}}}\,x \, < \, a} \\ {\left( {{\textstyle{{x \, - \, a} \over {t \, - \, a}}}} \right)^2} & {{{{{{{{\mathrm{for}}}}}}}}\,a \le x < \, t} \\ { - {\textstyle{1 \over 2}}\left( {{\textstyle{{b \, - \, x} \over {b \, - \, t}}}} \right)^2} & {{{{{{{{\mathrm{for}}}}}}}}\,t \le x \le b} \\ {} & {{{{{{{{\mathrm{for}}}}}}}}\,x \, > \, b} \end{array}} \right.$$
*off*	Similar to the “on” function but the step is downward from 1 to 0 as the first argument exceeds the threshold defined by the second argument. $off\left( {x,t} \right) = 1 - on\left( {x,t} \right)$

All functions return a unitless result in range 0–1.

## Results

### Predator-prey dynamics are highly replicable

Highly similar dynamics of flagellates and prey bacteria were observed in all replicate co-cultures, with very minor variation in amplitudes or timing (Fig. 1). In the absence of flagellates, bacteria grew exclusively as planktonic single-cells and reached a final steady-state abundance within a few days. By contrast, co-cultures of flagellates and bacteria exhibited marked drops and rises in abundances before the system eventually approached a steady-state about 4 weeks after inoculation. This steady-state was consistently characterized by the co-occurrence of a single-cell and a filamentous bacterial phenotype. Flagellate abundance stabilized at about 10^5^ cells mL^−1^ in the absence of marked predator-prey cycles. The system dynamics beyond day 6 were insensitive to initial predation pressure levels, which varied by two orders of magnitude (Fig. 1).

### Initial bacterial response to predation

In co-cultures, flagellates were highly successful in controlling bacterial densities until day 8 when the latter approached a level of about 10^6^ cells mL^−1^ (Fig. 1A). This was followed by a period of about four days where flagellate numbers declined sharply at a rate identical to the rate of dilution (0.5 day^−1^), indicating zero gross growth (Fig. 1C). This might appear at first glance to be an effect of food limitation commonly observed in cyclic predator-prey dynamics. However, the decline in flagellate numbers coincided with an immediate steep rise in bacterial abundance. Remarkably, predator numbers continued to decline beyond day 10 although prey bacteria had already recovered to 10^7^ cells mL^−1^, a level which perfectly supported flagellate growth earlier. Considering the observed flagellate generation times between 14 and 24 h, such a pronounced time lag between prey and predator recovery was not to be expected. Specifically, model simulations suggest a phase shift of about one day between predator and prey cycles in the absence of prey defense (Fig. S1); the observed recovery of flagellates, however, lagged behind the recovery of bacteria by 5–8 days.

**Fig. 3:**
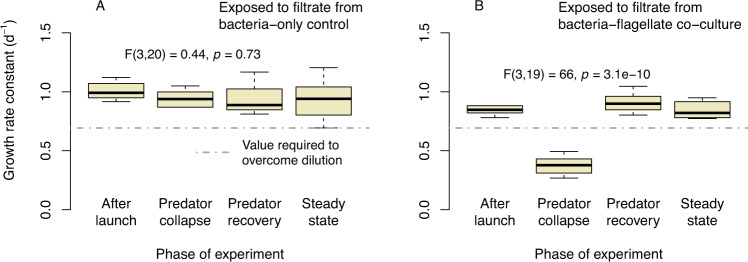
Growth rate constants of flagellates exposed to filtrate harvested from semi-continuous cultures during different phases of the experiment (cf. Fig. 1). **A** Filtrate of bacteria-only cultures, **B** filtrate of bacteria-flagellate co-cultures. Statistical information refers to one-way ANOVA. In (**B**), growth rates under exposure to filtrate from the phase of the predators’ collapse (group 2) were significantly reduced compared to any other phase (*p* < 0.001); differences between groups 1, 3 and 4 were insignificant (Tukey’s HSD test).

The hypothesis of predator inhibition was supported by acute toxicity tests in which axenically grown flagellates were exposed to sterile filtrate obtained from the semi-continuous cultures (Fig. 3). When exposed to filtrate from bacteria-only cultures, the growth rate of flagellates consistently exceeded the rate of dilution prevalent in the semi-continuous cultures without a significant dependence on the phase of the experiment when the filtrate was harvested (Fig. 3A). When confronted with filtrate from co-cultures, however, flagellate growth rates showed the same statistically significant drop as in the semi-continuous culture from which the filtrate was obtained (Fig. 3B).

### Convergence to final steady-state

After a short period with only minor changes in species’ abundances around day 13–16 (Fig. 1A, C), a quick recovery of flagellates was observed with their replication apparently no longer being compromised. The resulting predation pressure on bacteria translated into a marked decline in their numbers similar to earlier observations (Fig. 1, light blue scenario, day 3–8). However, the further dynamics deviated substantially from the previously observed pattern. In particular, repeated significant declines in predator abundance and recovery of prey bacteria did not occur. The abundances of flagellates and single-celled bacteria rather remained almost constant, while a filamentous bacterial phenotype emerged in detectable numbers around day 20 (Fig. 1B, D). On day 25, the biovolume of this filamentous phenotype was already on par with the biovolume of the previously predominant single-celled phenotype (about 1.5 × 10^6 ^µm mL^−1^ each). In subsequent days, filaments clearly established as the predominant phenotype in terms of biovolume with some individuals reaching up to 200 µm in length (Fig. S2). Considering the size of the predator, even much shorter filaments exceeding a size of about 5 µm can safely be regarded as grazing resistant [39]. This applies to over 97% of the observed individuals (Fig. S2).

The observed dynamics during the second half of the experiment certainly pose several questions: Why after all did *P. putida* not fall back to the earlier defense strategy, which had proven to efficiently suppress predation and supported an exceptionally high bacterial biomass around days 8-16? Was filamentation just an alternative induced defense?

Long-term cultivation of elongated *P. putida* isolates in the absence of predators demonstrated that filamentation was persistent. The vast majority of cells remained in filamentous shape and even single cells, formed by asymmetric division, continued to grow as filaments. This non-reversibility of filamentation pointed towards a genetic rather than an induced manifestation. Whole-genome sequencing of nine filamentous isolates from independent co-cultures finally revealed several non-synonymous base substitutions with respect to a single-celled control strain. In four out of nine isolates, these mutations were located in coding regions known to be involved in septum formation, namely in the genes *ftsQ*, *ftsA*, and *minC* (Table S1).

### No indication for adaptation of predators

**Fig. 4:**
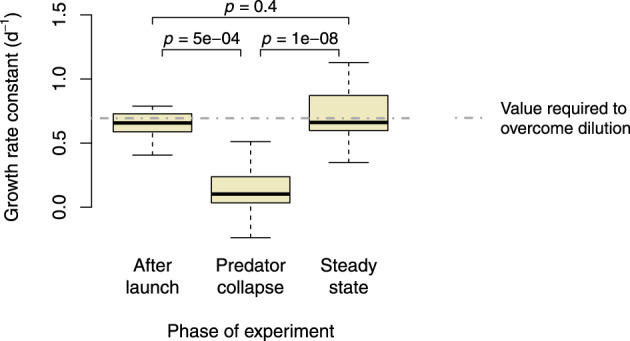
Growth rate constants of flagellates present in co-cultures at the onset of the filamentous steady state upon exposure to sterile filtrate harvested during earlier phases of the experiment. Labels under the boxes indicate the phase of filtrate harvesting. Adjusted *p* values refer to a Wilcoxon rank sum test.

In principle, the quick recovery of flagellates after day 16 could reflect the appearance and subsequent selection of a phenotype which is resistant to the toxic metabolites produced by *P. putida*. The emergence of an alternative bacterial defense like, e.g., filamentation could then be interpreted as a result of an ongoing evolutionary arms race [40]. However, auxiliary experiments did not provide any indication for adaptation of the flagellates to the bacterial toxin (Fig. 4). Flagellates present in co-cultures right after their recovery, at the onset of the filamentous phase, were still significantly inhibited in growth when confronted with sterile filtrate harvested during the period of the predator collapse (day 8–12). Even flagellates that were co-cultured with *P. putida* for more than 50 days remained susceptible to the toxic metabolites.

## Discussion

In co-culture with the bacterivorous flagellate *Poteriospumella lacustris*, the prey bacterium *Pseudomonas putida* exhibited a characteristic succession of predation defenses. The initial and the final defense differed substantially from one another with regard to their mechanism and their population-level benefits to the bacteria.

Our results strongly indicate that the initial bacterial defense falls into the category of chemical defense, and is regulated by phenotypic plasticity. This would require *P. putida* to be able to sense predator density and to regulate the excretion of inhibitory substances accordingly. Because a considerable proportion of the *P. putida* genome is known to be involved in regulation and signal transduction allowing for very flexible responses to environmental triggers [41] both conditions are likely to be met. The filtrate exposure tests (Fig. 3) provide specific evidence for the ability of *P. putida* KT2440 to up- and downregulate the excretion of compounds inhibiting flagellate growth in response to grazing pressure. Previous research [25] corroborated the ability of *P. putida* to escape grazing from bacterivorous flagellates through induced responses like aggregation or biofilm formation.

To provide a possible characterization for the apparent bacterial toxin, the whole-genome sequences of *P. putida* KT2440 obtained here were aligned against the antiSMASH [42] database. The output suggests the existence of non-ribosomal peptide synthetase clusters mediating the production of pyoverdines, a particular class of siderophores. The latter are molecules released by bacteria into the environment, which enhance the uptake of essential metals like, e.g., iron under deficient conditions. Specific pyoverdines associated with *P. putida* KT2440 have previously been identified [43]. Recent findings have shown that the benefits from siderophore production are not limited to competitive advantages gained from enhanced resource exploitation [44]. Pyoverdines were also demonstrated to determine the virulence of Pseudomonads via the damage of mitochondria in colonized hosts [45]. Moreover, pyoverdines were shown to be involved in the inducible defense of *P. putida* against predatory myxobacteria [46]. Such multiple functions have been reported for a number of bacterial metabolites, especially in Pseudomonads [47], and the particular combination of pyoverdin effects would explain the observed simultaneous flagellate inhibition and promoted bacterial growth.

In contrast to the initial chemical defense of *P. putida*, the subsequent filamentation clearly provides an example of rapid evolution. Although the responsible mutation(s) could only be pinpointed in a few isolates so far (Table S1), there is no doubt about the genetic manifestation and heritability of the filamentous phenotype due to its demonstrated non-reversible nature.

Only recently, similar observations were made by long-term co-cultivation of *Pseudomonas fluorescence* with the amoeboid predator *Neaglena grubei* [48]. In that system, protective adaptations like enhanced biofilm formation and altered motility were traced down to mutations in two particular genes (*wspF*, *amrZ*).

From the perspective of the bacterial population, filamentation appears to be a much less efficient defense mechanism than toxin production. This is clearly reflected by the ratio of prey to predator biomass, which differed by two orders of magnitude between the initial and final defense (Table 6). It raises the question of why bacteria would abandon a highly effective form of defense in favor of a much less effective one. As demonstrated experimentally, adaptation of predators to the toxin can be excluded as a cause (Fig. 4). Moreover, it was not instantly evident how the small-sized flagellate was ultimately able to persist in large numbers given a very high proportion of completely inedible prey individuals (Fig. 1D and Fig. S2).

**Table 6:** Average abundance of predator and prey during the temporary steady state following the initial bacterial defense (day 13–16) and during the final steady state (beyond day 30).

	Day 13-16	Beyond day 30
Flagellates (cells mL^−1^)	2000	80,000
Single-celled bacteria (cells mL^−1^)	7 * 10^7^	5 * 10^6^
Filamentous bacteria (cell equivalents mL^−1^)	0	2 * 10^7^
Total bacteria (cell equivalents mL^−1^)	7 * 10^7^	2.5 * 10^7^
Prey to predator ratio	35,000	313

Cell dimensions did not vary substantially over time and cell counts are thus representative of the respective biomasses. For filaments, 1 µm was counted as 1 single-cell equivalent (cf. Fig. 1D).

To develop a comprehensive understanding of the system addressing the questions raised above, we set up a semi-continuous differential equation model to simulate the dynamics of predator and prey phenotypes. The model considers seven state variables (carbon, densities of four bacterial phenotypes, flagellate density, and toxin concentration) whose dynamics are controlled by nine processes (Table 3, Fig. 2). In addition to microbial growth and grazing, the model implements a phenotypically plastic predation defense (toxin production) as well as a genetic defense (filamentation) which arises via mutation. The particular assumptions implemented in the model are as follows:

### Dual effect of bacterial metabolites

In line with the above discussion on siderophore-like compounds, secondary metabolites excreted by *P. putida* were assumed to exhibit a dual function, both inhibiting the growth of flagellates and allowing for a more efficient exploitation of the resources by bacteria. The inhibition of predators was demonstrated directly (Figs. 3 and 4) while enhanced resource exploitation was inferred from bacterial abundances in co-cultures exceeding the carrying capacity observed in predator-free controls (Fig. 1A, day 11–18).

### Metabolite production is costly

The production of bacterial metabolites was assumed to be associated with a slight fitness cost [49] since resources are diverted from reproduction, thus resulting in a lowered growth rate of toxin-producing bacteria. The assumed fitness cost of 11% (parameter *cBx* in Table 5) allowed for the best agreement between simulated and observed data and is in agreement with data on the cost of pyoverdine production by *P. aeruginosa* [50]. The cost only manifests when toxin production is upregulated.

### Predator recognition and quorum sensing interact

In the model, the production of bacterial metabolites is upregulated when the two conditions of high flagellate abundance and high bacterial abundance coincide. That is, the expression of the toxin-based bacterial defense is assumed to be jointly controlled by predator recognition and quorum sensing (QS). Examples for such joint control of bacterial defenses have been reported previously [8, 26, 51]. The involvement of QS in chemical defense strategies is particularly likely as effective toxin concentrations can only be reached when producers are highly abundant. While multiple QS systems have been described for other Pseudomonads, only a single system has been identified in *P. putida* KT2440 so far [52, 53].

### Mutation rates are conditional on stress

The emergence of mutations resulting in the filamentation of *P. putida* was assumed to be conditional on a high ambient concentration of bacterial metabolites. The latter was considered as a proxy for bacterial stress which can affect mutagenesis either directly or indirectly by a variety of mechanisms [54–56]. Without this assumption, the almost synchronous appearance of filaments in all replicates at a late point in time would be very difficult to explain. Specifically, if mutation frequencies were high, filaments would become the predominant phenotype early (Fig. S3) which contradicts observations. On the other hand, if frequencies were low but unconditional, the timing of filament appearance should vary between replicates, which is in contrast to observations either (Fig. 1B).

### Filamentation is associated with a fitness cost

Measurements of growth rate constants revealed a significant fitness disadvantage of filamentous isolates in comparison to single-celled, undefended isolates (*p* < 0.05, Wilcoxon rank sum test). Under the given experimental conditions (wheat grass medium, 19 °C) the growth rate of filaments was 5% lower than the growth rate of single-celled, undefended competitors.

### Filaments do not produce metabolites at toxic levels

When flagellates were exposed to sterile filtrate harvested from cultures in the filamentous stage, no inhibition of growth was observed (Figs. 3 and 4). Owing to this empirical result and the questionable benefit of a redundant defense, the model does not consider metabolite production by filaments.

### Filaments undergo asymmetric division

Upon growth, bacterial filaments are assumed to divide regularly at their tips [57]. Specifically, the model is built on the assumption that elongation occurs with a certain probability *p* while single-celled, undefended offspring are produced with probability *1-p*. Division is obviously necessary for filaments to persist in liquid cultures subject to continuous dilution.

**Fig. 5:**
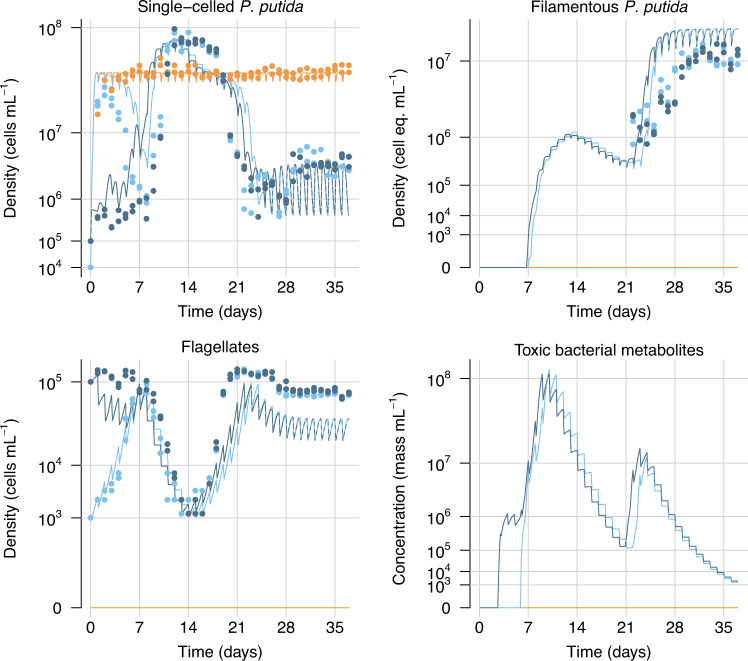
Simulated dynamics of selected state variables (lines) in comparison to observations (dots). Colors indicate different initial values of flagellate abundance (see legend of Fig. 1).

The model was able to qualitatively and quantitatively reproduce all major patterns of the observed predator-prey dynamics (Fig. 5), confirming that the above set of assumptions are a plausible explanation for the observed experimental dynamics. In particular, the model correctly predicted the shift from toxin production, resulting in a high-amplitude cyclic dynamic, to a stable steady state where filamentous bacteria are the predominant phenotype and toxin production has been abandoned. But the real strength of the model lies in the opportunity to analyze the specific contributions of individual processes which can hardly be disentangled from empirical observations alone. The simulations offer an explanation for the persistence of high predator numbers in a world of oversized, inedible prey: the flagellate numbers are mainly supported by the continuous release of single cells during asymmetric filament division (Fig. S4).

The model also confirms that the filamentous genotype only emerges during the experiment and could not be just a result of selection from a heterogeneous inoculum. Any tiny seed of mutants provided to the model would result in the early predominance of filaments in simulated co-cultures (Fig. S3). Furthermore, the model predicts the emergence of filamentation as the final bacterial defense strategy, even when it was assumed to come at a much higher cost than toxin production (Fig. S5) and despite this strategy being far less efficient from a population point of view (Table 6). This counter-intuitive result can be explained by the fact that selection acts at the level of individuals and does not necessarily maximize population fitness [58]. Thus, the ultimately superior strategy is determined by the fitness of filaments in comparison to the fitness of toxin-producers in a mixed population. This, in turn, depends on the relative costs and benefits of each defense strategy. The model results make it clear that filamentation is the superior strategy due to its relative benefit, i.e. the level of grazing protection (Fig. S6). Excretion of toxins represents a concerted action of the bacterial community mediated by cell-to-cell signaling which benefits the population as a whole, acting as a public good [17]. In contrast, filamentation provides grazing protection only to the individuals carrying this trait and cannot be exploited by non-filamentous individuals. This distinction is crucial for determining the outcome of selection: since filamentous bacteria benefit from both forms of defense, they always have a stronger grazing protection than toxin-producing bacteria, which can offset even relatively high costs.

In addition to its higher fitness, other factors are likely to further promote the persistence of filaments. First, owing to its genetic manifestation, the filament-based protection is unconditionally stable whereas induced defenses undergo reversal when trigger conditions are, even temporarily, no longer met. It has been suggested that inducible defenses may be particularly favorable precisely because they can be switched off when they are not needed, thus allowing the individual to economize on the costs of defense [59]. However, the reversible nature of the initial defense appears to be a disadvantage in our system, as it allows the recovery of the flagellates to high density and thereby sets the stage for the inedible filamentous bacteria to become dominant. Second, there is a self-stabilizing effect associated with the filamentous phenotype. In the late phase of the experiment, the majority of the available resources are consumed by the filamentous bacterial genotype (Fig. S4), preventing undefended single cells from reaching the critical density required to trigger the initial, community-driven defense once again.

Unlike individual-based strategies, community defenses based on public goods are vulnerable to “cheating” [17] in the sense that a sub-population emerges that benefits from cooperation but does not pay the costs. Siderophore production in *Pseudomonas aeruginosa* is a classic model system for resistance to cheating and the maintenance of cooperation [15, 60]. The results of these previous studies suggest that, in our experimental system, due to the lack of spatial structure which may promote the maintenance of cooperation [15, 17, 61], non-toxin-producing cheaters would eventually displace toxin-producers. However, additional model simulations showed that the observed breakdown of the community defense in our experiments is unlikely to be a result of cheating. Specifically, model simulations that incorporate the emergence of a cheater mutant indicate that, while such cheaters will inevitably rise to dominance and cause the collapse of cooperative toxin production, this will take far longer than the rapid dominance of filamentous bacteria (Fig. S7) because the fitness advantage of cheaters is relatively small. This finding provides a highly relevant new angle to the question of the maintenance of cooperation, which has been extensively investigated with a focus on resistance against cheating. Our results indicate that cooperation may be far more vulnerable to the evolutionary invasion of individual-level strategies that provide the same benefit.

Overall, the unique succession of bacterial defenses observed in our long-term co-cultures demonstrates that inducible traits and rapid evolution not only impact population dynamics but also interact with each other. In particular, our results demonstrate that an inducible defense can pave the way for an evolved defense, which in turn suppresses the original one.

## Supplementary information

Supplement

## Data Availability

Sequence data generated during the current study have been uploaded to the NCBI SRA database with accession number PRJNA830374. A ready-to-run version of the mathematical model, including the complete source code, as well as time-resolved data on organisms counts were deposited at https://github.com/dkneis/KT2440defense for uncomplicated accessibility.
