# Supplementary material for: Shifts from cooperative to individual-based predation defense determine microbial predator-prey dynamics
Source: ISME J. 2023 Feb 28;17(5):775–85. doi: 10.1038/s41396-023-01381-5 (PMC10119117; doi:10.1038/s41396-023-01381-5)
Supplement: Supplementary file 1 — Supplement [file 41396_2023_1381_MOESM1_ESM.pdf]

Shifts from cooperative to individual-based predation defense determine microbial predator-prey dynamics

Magali de la Cruz Barron[1,2], Ellen van Velzen[3], Uli Klümper[2], Markus Weitere[1],
Thomas U. Berendonk[2], David Kneis[2*]

1 Helmholtz Centre for Environmental Research - UFZ, Department of River Ecology, 39114 Magdeburg, Germany
2 TU Dresden, Institute of Hydrobiology, 01062 Dresden, Germany
3 University of Potsdam, Institute of Biology and Biochemistry, 14469 Potsdam, Germany

* david.kneis@tu-dresden.de

Main article DOI: 10.1038/s41396-023-01381-5

Supplementary material

Mutations identified in filamentous *P. putida*

Table S1: Single nucleotide variations in cell division-related genes found in filamentous isolates of *P. putida* (n=9) with reference to a single-celled control strain.

Isolate ID	Position in Genome	Base switch	Effect	Gene	Gene function
7, 9	1526945	G → A	Gly → Asp	*ftsQ*	Cell division protein
8	1528549	T → C	Val → Gly	*ftsA*	Cell division protein
5	1934576	G → A	Val → Ile	*minC*	Septum-site determining protein

Expected phase shift in cycles of predator and undefended prey

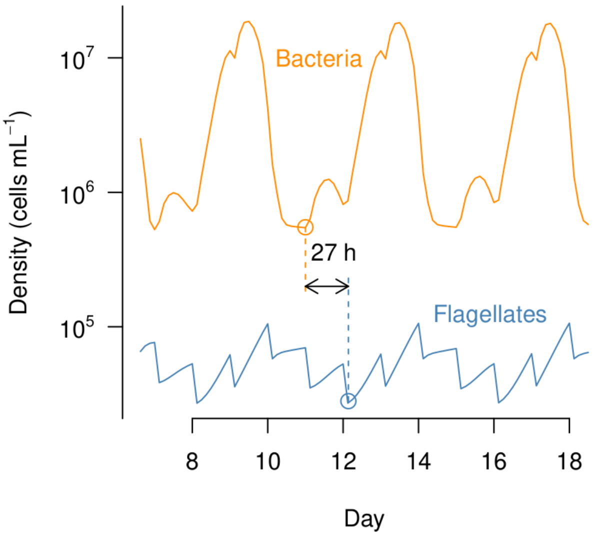

Figure S1: Simulated dynamics of prey and predator density for a scenario where bacteria do not develop any defense. The arrow indicates the phase shift between cycles with regard to the respective minimum values.

Length distribution of bacterial filaments

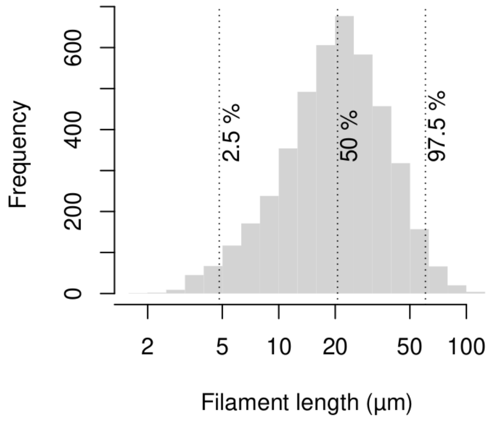

Figure S2: Histogram of bacterial filament lengths observed in the late phase of the experiment beyond day 30. Dotted lines indicate quantiles.

Predicted dynamics when the inoculum contains filamentous mutants

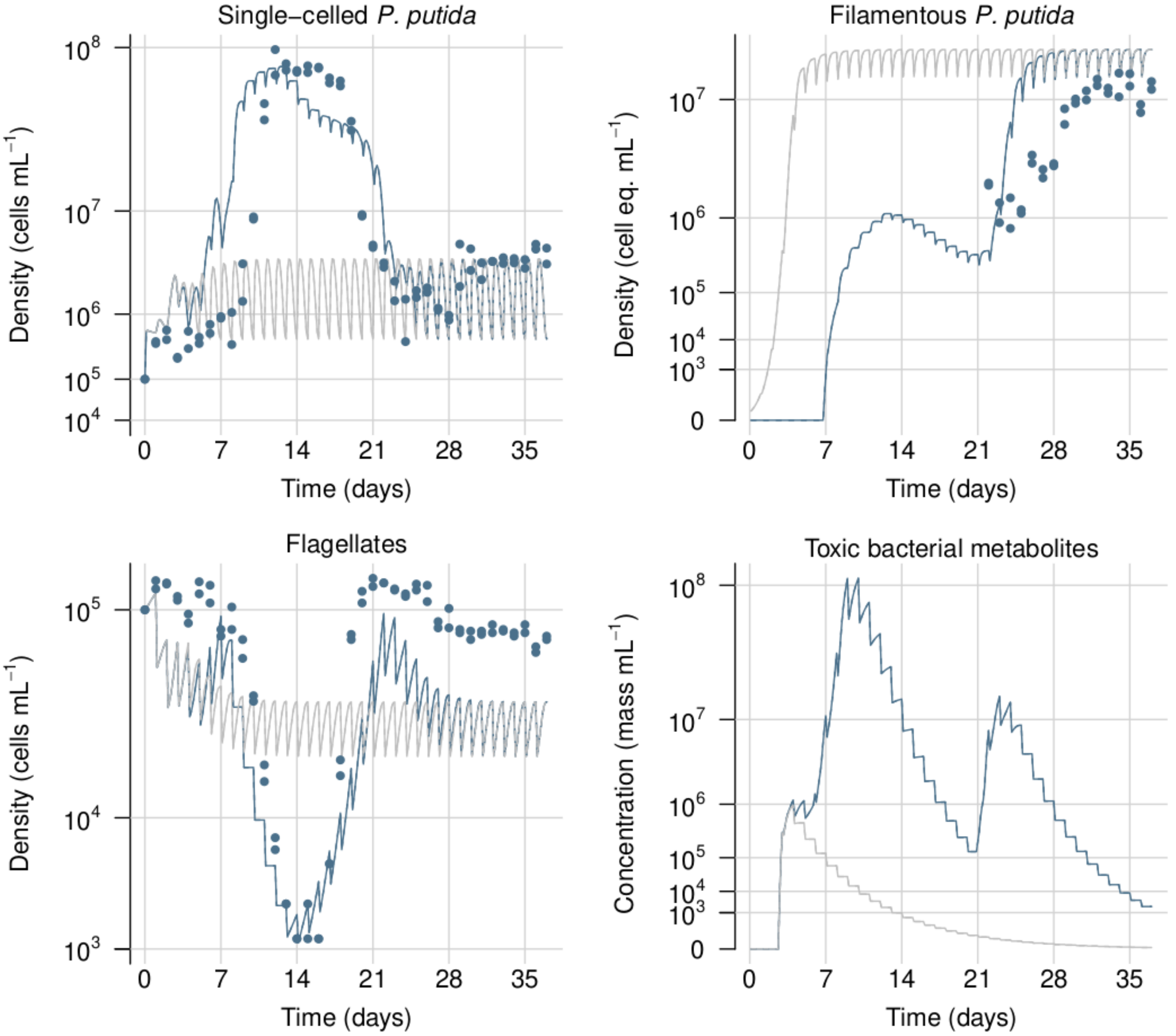

Figure S3: Predicted effect of the presence of the filamentous genotype in the inoculum. The blue scenario with corresponding observations is the same as in Fig. 5. Gray graphs illustrate the predicted dynamics when filaments are present initially in very low abundance (1 single-cell equivalent / 10 mL^{-1}).

Decomposition of simulated bacterial dynamics

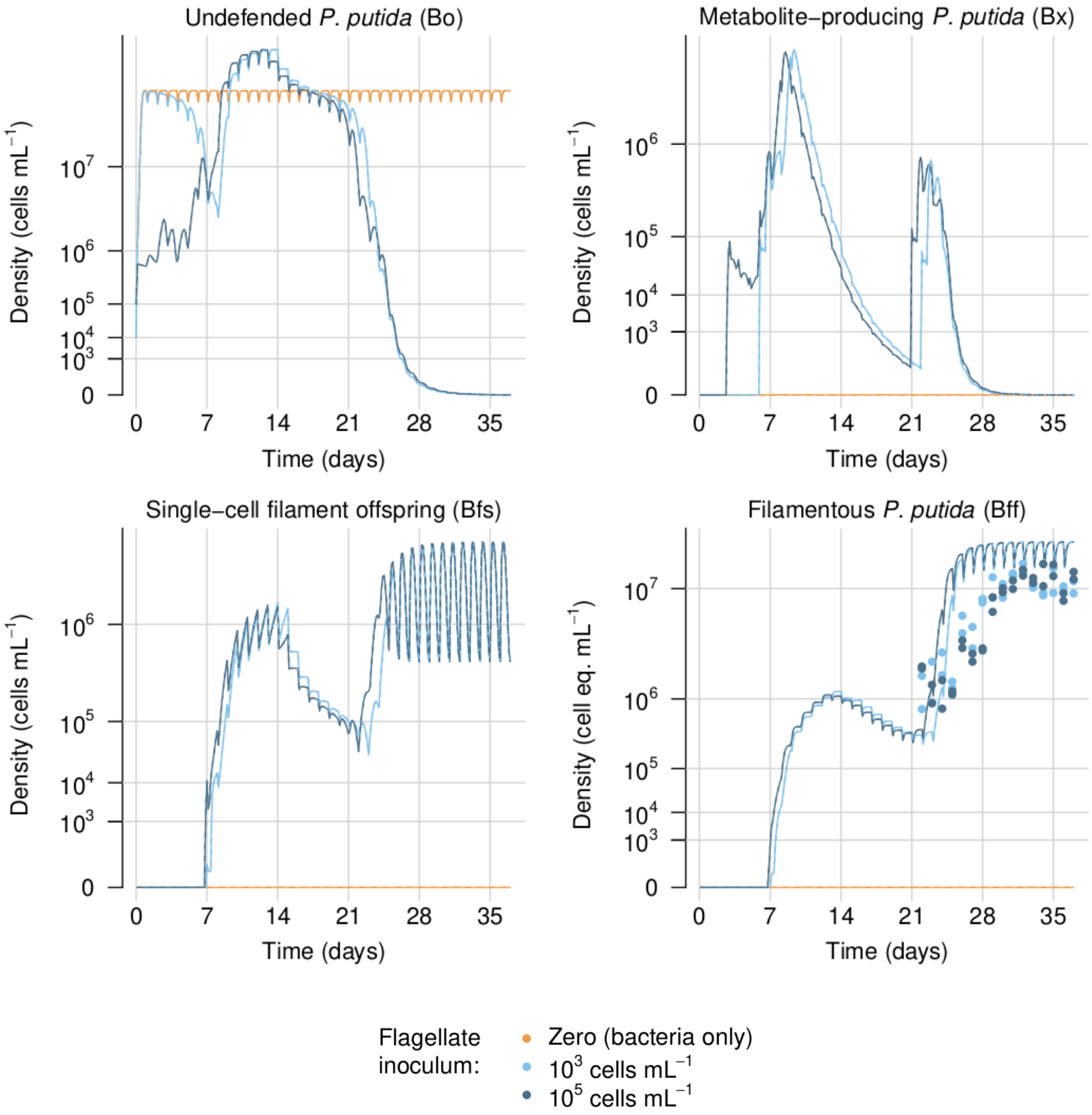

Figure S4: Decomposition of the simulated bacterial dynamics displayed in the top panel of Fig. 5. In particular, single-celled bacteria addressed by the top-left plot of Fig. 5 were decomposed into the three distinct fractions *Bo* (undefended), *Bx* (metabolite-producers), and *Bfs* (single-celled filament offspring). According to simulation, beyond day 25, the vast majority of single-celled bacteria belongs to the *Bfs* fraction (sub-figure at bottom left). Thus, high flagellate densities during the late phase of the experiments were essentially maintained by asymmetric filament division.

Additional simulation results

To elucidate why filamentous bacteria become dominant over single-celled toxin-producing bacteria, and to determine how sensitive this result is to the costliness of filamentation compared to the costliness of toxin production, we ran additional simulations. In those, we varied the cost of filamentation and performed a more detailed analysis on the results.

These simulations show that the eventual dominance of filamentous bacteria is highly robust: the simulated dynamics are almost identical even if the growth rate of filamentous bacteria was reduced by 40% (Fig. S5). For comparison, toxin production carries a growth cost of 11% (Table 5).

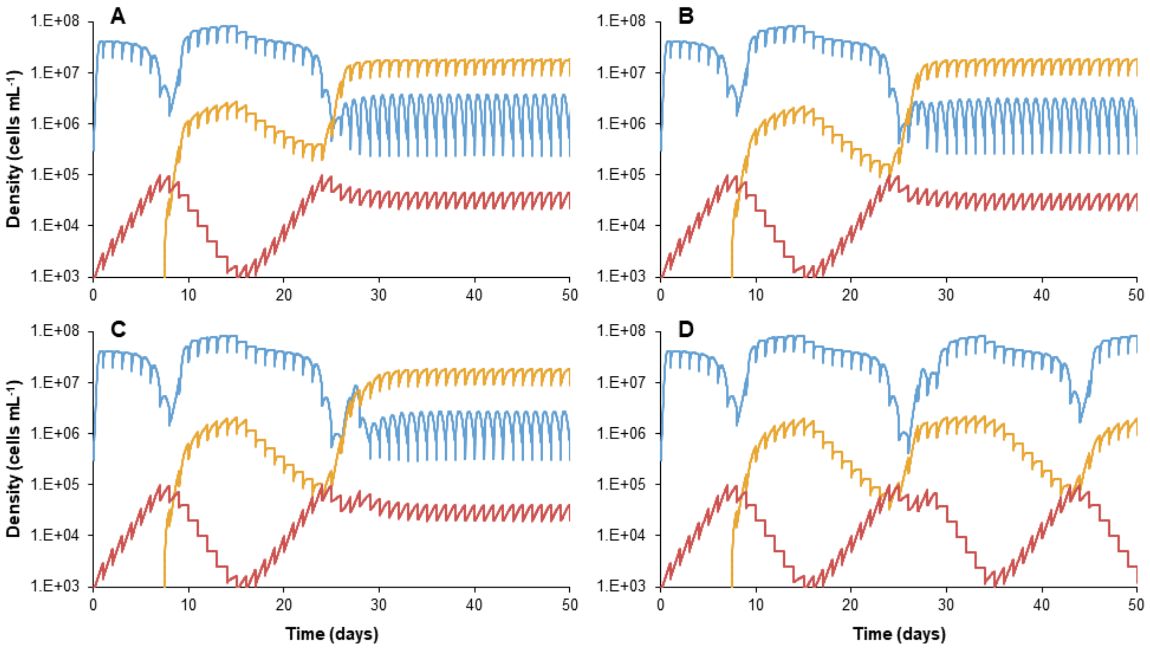

Figure S5: Additional simulation runs varying the costliness of filamentation; all other parameters have values found in Table 5. A: growth reduction by 10%; B: 30%; C: 40%; D: 50%. Blue lines: single-celled bacteria; orange lines: filamentous bacteria; red lines: flagellates.

The reason behind this result can be found in the relative costs (measured by the growth rates) and benefits (measured by the predation rates) of the two strategies. Despite being much less efficient on a population level, filamentation is a highly effective form of defense on an individual level, as the grazing risk of filamentous bacteria is strongly reduced compared to that of toxin-producing bacteria (Fig. S6 A-C, dashed lines). This benefit is so strong that it can outweigh even a severe growth cost (Fig. S6 A-C, solid lines), and allows the filamentous bacteria to quickly rise to dominance unless the cost is very high (as seen in Fig. S5 D).

Interestingly, if filamentation comes at a cost, it is always inferior to the toxin-producing strategy when toxin concentration is high enough to inhibit flagellate growth (days 10-15 in all panels of Fig. S6). This is because, while all bacteria benefit from the toxin-induced grazing inhibition, it actually removes the advantage that filamentous bacteria have over toxin-producing bacteria. From a

competitive viewpoint, filamentous bacteria benefit most strongly from high grazing pressure combined with a lack of toxin production (days 30-40 in Fig. S6 A-C), as this combination leaves the non-filamentous bacteria completely vulnerable while the filamentous bacteria benefit from their grazing protection. This generates a self-stabilizing effect of filamentation: because the majority of the available resources accumulates in bacterial filaments, the density of single-celled bacteria remains too low to trigger toxin production; this, in turn, keeps predator density high, which causes the filamentous bacteria to maintain their advantage.

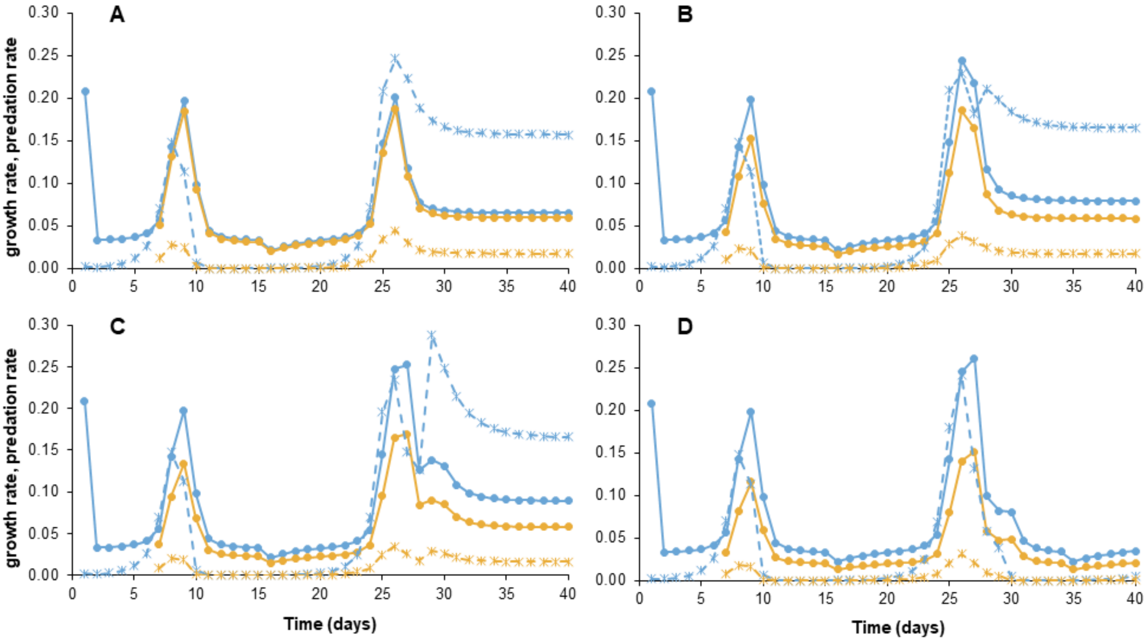

Figure S6: Per capita growth rates and predation rates belonging to the simulated dynamics in Fig. S5. Growth and predation rates of the "toxin-producing" strategy (*Bo* + *Bx*) are shown in light blue, those of the "filamentation" strategy (*Bfs* + *Bff*) in orange. Solid lines and filled symbols represent growth rates; dashed lines and crosses represent predation rates. Values represent the average for each day for clarity of presentation; note that days 1-6 do not contain values for the filamentation strategy, as this only emerges on day 7.

Finally, we modeled a hypothetical scenario where a "cheater" mutant arises: a genotype where upregulation of toxin production is suppressed, which allows them to benefit from the toxins produced by others without having to carry the cost themselves. It is well known that, without additional factors such as spatial structure, such cheaters will always have a higher fitness, and this is indeed reflected in the simulated dynamics (Fig. S7). However, it is notable that while the cheaters clearly have an advantage over the original toxin-producing strategy, their advantage is not nearly as strong as that of the filamentous bacteria. While filaments typically rise to dominance before day 30 (Fig. S5), cheaters remain outnumbered by non-cheaters after 100 days (Fig. S7). Clearly, losing the costs of defense is not nearly as advantageous as gaining an additional form of grazing protection, even when this extra protection comes at a substantial cost.

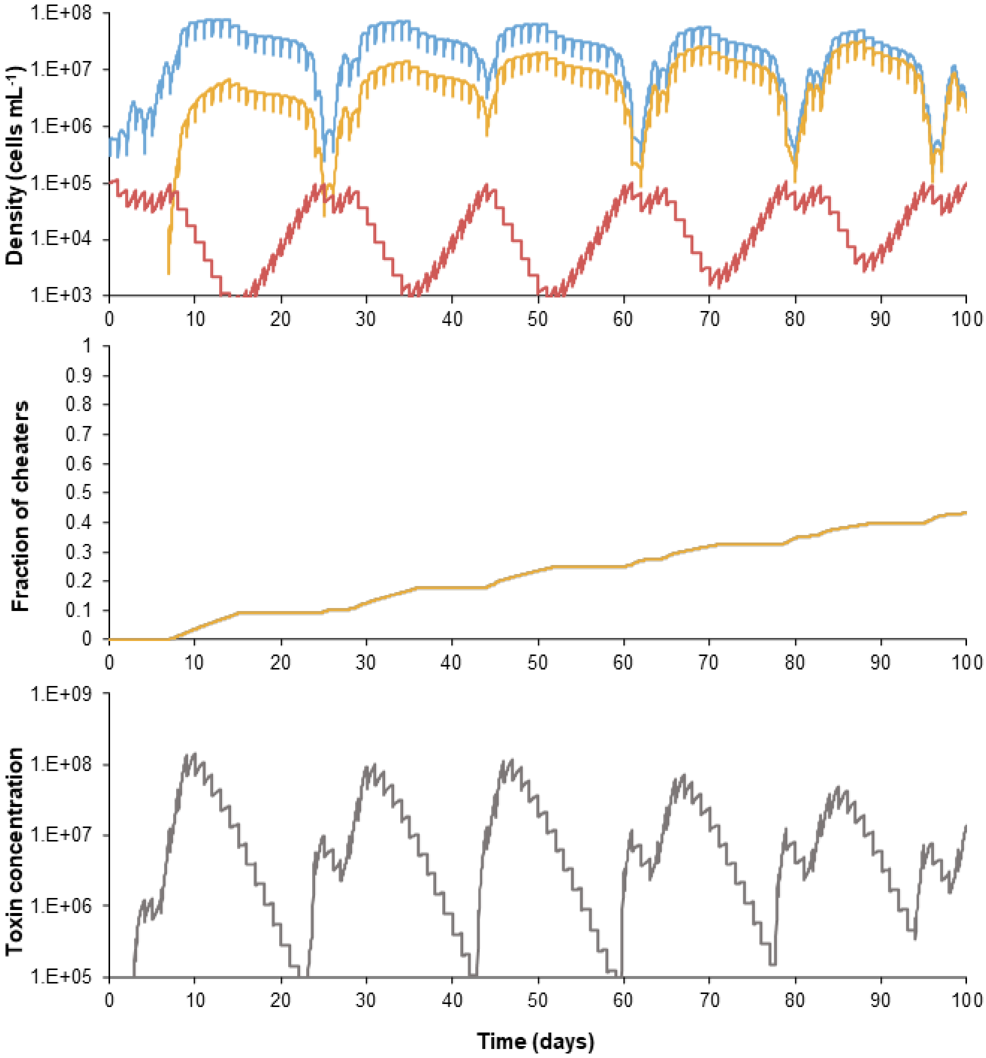

Figure S7: Simulation run showing the emergence and rise of a hypothetical "cheater" mutant, which is identical to the original "single-celled, non-toxin-producing" bacteria Bo but never upregulates toxin production. Top panel: densities of the original bacteria (blue), cheaters (orange) and flagellates (red). Middle panel: fraction of cheaters in the total bacterial population. Bottom panel: toxin concentration.
